# Tissue-specific proteome profile analysis reveals regulatory and stress responsive networks in passion fruit during storage

**DOI:** 10.1038/s41598-024-52557-8

**Published:** 2024-02-12

**Authors:** Ellen Garcia, Jin Koh, Xingbo Wu, Ali Sarkhosh, Tie Liu

**Affiliations:** 1 Horticultural Sciences Department, University of Florida, Gainesville, FL 32611 USA; 2 The Interdisciplinary Center for Biotechnology Research, University of Florida, Gainesville, FL 32611 USA; 3 Department of Environmental Horticulture, Tropical Research and Education Center, University of Florida, Homestead, FL 33031 USA

**Keywords:** Agricultural genetics, Biotechnology, Plant sciences

## Abstract

*Passiflora edulis*, commonly known as passion fruit, is a crop with a fragrant aroma and refreshingly tropical flavor that is a valuable source of antioxidants. It offers a unique opportunity for growers because of its adaptability to tropical and subtropical climates. Passion fruit can be sold in the fresh market or used in value-added products, but its postharvest shelf life has not been well-researched, nor have superior cultivars been well-developed. Understanding the proteins expressed at the tissue level during the postharvest stage can help improve fruit quality and extend shelf life. In this study, we carried out comparative proteomics analysis on four passion fruit tissues, the epicarp, mesocarp, endocarp, and pulp, using multiplexed isobaric tandem mass tag (TMT) labeling quantitation. A total of 3352 proteins were identified, including 295 differentially expressed proteins (DEPs). Of these DEPs, 213 showed a fold increase greater than 1.45 (50 proteins) or a fold decrease less than 0.45 (163 proteins) with different patterns among tissue types. Among the DEPs, there were proteins expressed with functions in oxygen scavenging, lipid peroxidation, response to heat stress, and pathogen resistance. Thirty-six proteins were designated as hypothetical proteins were characterized for potential functions in immunity, cell structure, homeostasis, stress response, protein metabolism and miraculin biosynthesis. This research provides insight into tissue-specific pathways that can be further studied within fruit physiology and postharvest shelf life to aid in implementing effective plant breeding programs. Knowing the tissue-specific function of fruit is essential for improving fruit quality, developing new varieties, identifying health benefits, and optimizing processing techniques.

## Introduction

Passion fruit is a climacteric fruit with high metabolic activities after harvest^1^ and is grown in many tropical and subtropical countries where it is a popular commodity. The pulp has a high nutritional and phytochemical value, containing vitamins A and C, zinc, and magnesium as well as polyphenols and carotenoids^2^.Some health benefits of passion fruit have been found in vitro for antioxidant, anti-inflammatory, antibacterial, and antifungal activities, and in vivo, such as the management of diseases including asthma, hypertension, osteoarthritis, diabetes, and pulmonary fibrosis^3^. Markets for the fruit include fresh fruit, processed goods, nutritional supplements, and cosmetic products.

The purple variety of passion fruit is especially prone to rapid deterioration due to water loss and cell wall deterioration, resulting in a wrinkled fruit that is highly susceptible to pathogens and unmarketable, even when the edible portion remains unchanged in quality^4^.One of the major limiting factors for increasing the potential of the passion fruit market in the United States is the shelf life, which is about 8–10 days at room temperature, limiting transportation, marketability, and crop value^5^. Physiological changes that occur after harvest and lead to senescence include water loss, cell wall degradation (cellulose, hemicellulose, and pectin), solute transport, lipid metabolism, protein transport, hormone changes, sugar metabolism, reactive oxygen species accumulation, and antioxidant activity^6^. Therefore, finding effective strategies for preserving fruit quality and delaying deterioration in passion fruit is of paramount importance for both growers and consumers. However, there is an urgent need for molecular data to further investigate the biological mechanisms critical for passion fruit maturation at the molecular level.

Tissue-specific research can be used to compare different parts of the entire plant, such as source-sink relationships^7^ and how this effects the partitioning of carbohydrates^8^, the specificity of transporters in different tissue types^9,10^, and the spatiotemporal regulation of secondary metabolite biosynthesis and sucrose synthesis regulation that is promoter specific in different tissue types^9,10^. More research is needed to better understand the underlying mechanisms behind the ability of different tissues to contain highly specific proteins and how these fundamental differences are determined at the cellular level. This knowledge would allow breeding of novel crops as well as the specific targeting of genes within certain cell types or tissues in transgenic plants, potentially minimizing off-target or unintended effects.

Multi-omics techniques have been used extensively to study many aspects of fruit development, ripening, and stress responses, resulting in the identification of biomarkers linked to postharvest changes and physiological responses in important crops such as bananas, strawberries, blueberries, orange, and peaches^11^. Proteomics studies are vital because expression can be regulated post-transcriptionally and a gene transcript can make a variety of proteins through alternative splicing or produce many similar proteins, or isoforms, with potentially differentiated functions. It is important to study these to better understand what is happening at the cellular level and how these isoforms may function differently between tissue types. Genetic responses to environmental cues or cues within a cell can alter gene expression and further alter protein production, ultimately leading to physiological changes.

Currently, the proteins expressed in passion fruit are found through data mining of the genomes^12,13^, however, there is insufficient data on quantification of proteins and in various tissues. A proteome analysis of purple passion fruit is limited to two studies on the leaves using a 2D gel technique^14^ and mass spectrometry used for analysis in response to the cowpea aphid-born mosaic virus that identified approximately 500 proteins^15^. Therefore, the contribution of this study is the assessment of a larger-scale protein database with quantification of abundances in 3 distinct skin tissues of passion fruit. The realistic applications of this research are to enable the development of protein inhibitors, providing candidate genes for gene editing technology, or developing spray coatings or packaging that will allow for extending the shelf life of passion fruit using the differentially expressed proteins that warrant further investigation.

We identified key pathways in passion fruit for unveiling stress responses and shelf life. These pathways that can be studied further include heat shock proteins that are involved in the endoplasmic reticulum associated degradation, lipid transfer protein activity, apoptosis ferroptosis, and the role of antioxidants oxygen scavenging abilities in shelf life and pathogen resistance. The unexpected findings include the identification of potential novel proteins that involve in the unknown miraculin pathway, the role of zinc transporters in degradation processes, hormone pathways, and cell wall metabolism process. Notably, the investigation identified the major allergen pru ar 1, a crucial protein influencing both consumption and serving as a defense mechanism in passion fruit, peach and other plants, opening up a new avenue in biological exploration. This paper highlights the key proteins and pathways that can be targeted to better understand their role in the ripening process. There is an existing gap in understanding fruit ripening and senescence and to a greater extent how this works in passion fruit specifically. This research fits into the broader context of this field with implications for postharvest practices and future research directions.

Postharvest physiology and ripening in passion fruit are not well documented, which is an impediment to designing improved postharvest handling and storage regimes. This includes the postharvest management which is defined as the methods and techniques applied to increase the shelf life during postharvest activities such as harvesting, handling, and storage from the time of harvest up until the time and place of consumption^16^. A better understanding of postharvest physiology can relieve the current constraints on shipping passion fruit to distant markets. In this study, the proteome of distinct tissues in passion fruit were analyzed after harvest using tandem mass tagging (TMT) for quantitative proteomics. The results identified (1) proteins related to the progression of ripening and decay, (2) regulatory and stress responsive networks, and (3) the hypothetical proteins serving various functions. Understanding the functions of these proteins and metabolic pathways in passion fruit could potentially lead to the development of new technologies that reduce the significant loss of revenue during postharvest harvesting, handling, storage, and transportation.

## Results and discussion

### Proteomic analysis of passion fruit tissues reveals diverse functional signatures

**Figure 1:**
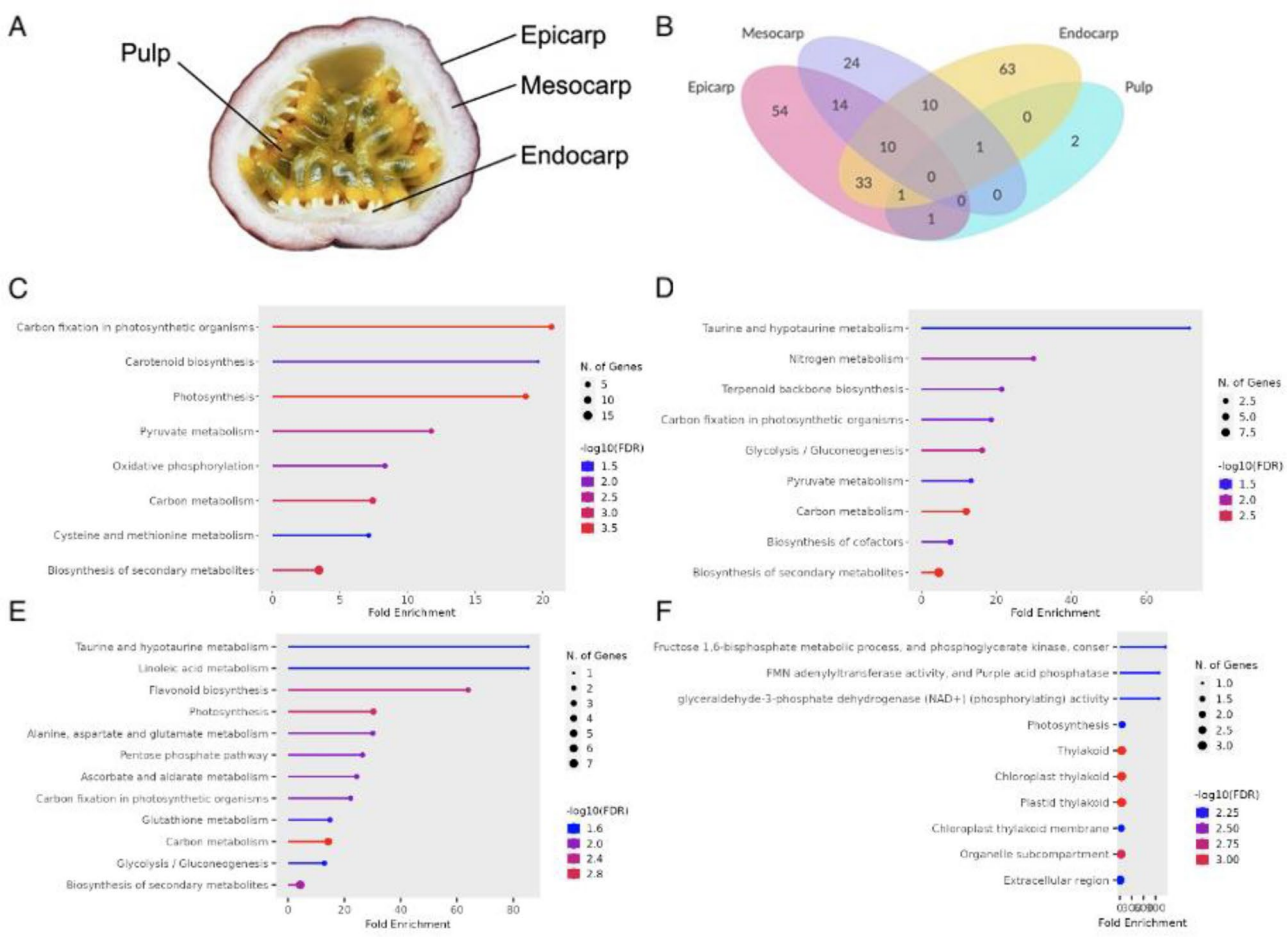
Passion fruit tissue types and differentially expressed proteins (DEPs) within each tissue. (**A**) internal distinction of passion fruit tissue types and (**B**) Venn diagram showing DEPs unique to each tissue type as well as those in common. The gene ontology (GO) terms for the differentially expressed proteins within each tissue type are shown for the enriched pathways in the epicarp (**C**), mesocarp (**D**), endocarp (**E**), and pulp (**F**). Significantly enriched GO terms were selected based on FDR < 0.05.

The proteomes of four postharvest passion fruit tissues, the epicarp, mesocarp, endocarp, and pulp, were analyzed, and protein levels in the different tissues were quantified (Fig. 1A). TMT labeling-based quantitative proteomics was carried out including independent biological replicates as described in the materials and methods. A total of 3352 proteins were identified. The expression patterns of these proteins in different tissues are shown in a Venn diagram (Fig. 1B) and Supplementary Table S1 in the Supporting Information. The Gene Ontology (GO) database was used to categorize all the identified proteins, which cover a wide range of biological processes, cellular components, and molecular functions (Supplementary Table S1). The top two dominant terms were ‘cellular anatomical entity’ and ‘intracellular anatomical structure’ in a cellular component, ‘catalytic activity’ and ‘hydrolase activity’ in molecular function, and ‘metabolic process’ and ‘organic substance metabolic process’ in biological process in the entire data set (Supplementary Table S1).

### Similarities between tissue types reveals key processes in storage: lipid transfer process, senescence associated gene, and photosynthesis

Out of the 3352 total proteins identified in all four tissue types (Fig. 1A,B), 295 proteins were determined to be differentially expressed in at least one tissue. None of the differentially expressed proteins (DEPs) were shared between the four tissue types. Several proteins were shared between tissue types, especially between the three fruit skin layers. Only a few proteins were present in the pulp and shared with one other layer (Fig. 1B). There were 54 DEPs unique to the epicarp, 24 DEPs unique to the mesocarp, 63 DEPs unique to the endocarp, and 2 DEPs unique to the pulp.

Ten proteins were shared between all three skin layers (epicarp, mesocarp and endocarp), and three of them are the lipid transfer proteins LTP1, LTP10, and LTP5. The LTP genes have been reported to regulate disease severity and play a role in salt stress in *Arabidopsis thaliana*^17^. Lipid transfer proteins play an important role in the fruit ripening process. A recent study found that the LTP genes were expressed in two organelles at the same time, forming a shuttle, bridge, or tube that links the donor and acceptor compartments, indicating its important role in lipid transportation and distribution^18^.

The cysteine protease senescence-associated gene 12 (SAG12) was differentially expressed in all three skin tissues but was upregulated in the epicarp and downregulated in the meso- and endocarps. There is limited research on the role of SAG12 in senescence of fruit; however, it is a senescence-specific cysteine protease that was reported to be significantly expressed in fruit under stress conditions, with a resulting decrease in yield and fruit weight^19^.

The heat shock protein HSP22 was the only protein upregulated in the mesocarp. In addition, it was downregulated in the epicarp and endocarp. In peas, HSP22 is a small mitochondrial heat-shock protein that serves as a thermosoluble chaperone that is prone to co-precipitating with unfolded client proteins under heat stress. It is induced by oxidative stress and heat during seed development. HSP22 can also function as a holdase that prevents the aggregation of some proteins while co-precipitating with others to facilitate their subsequent refolding by disaggregates or their clearance by proteases^20^. Heat shock proteins may be involved in cell wall remodeling and promoting mechanisms to resist heat stress using thermomemory. The involvement of plant cell wall remodeling in thermometry is not widely researched, but it is suggested by both the upregulation of genes encoding plant cell wall proteins by the heat inducible transcription factor HSFA2 as well as the cell wall proteins BIIDXI and DUF642, which regulate pectin methyl esterase activity^21^.

Other proteins identified in these three skin tissues were glutamate decarboxylase 5 (GAD5), DEFIN (MCB22), chloroplastic β-carbonic anhydrase (β-CA), AT3g18860, and chloroplastic thiamine thiazole synthase (THI). These proteins are factors in the ripening and senescence of all three skin layers and may be potential targets for prolonging shelf life. These proteins may play an important role by interacting with activators or repressors of the senescence process.

From the pulp, one protein was shared with the epicarp and one with the mesocarp and endocarp. The former was the glyceraldehyde-3-phosphate dehydrogenase GAPB, which increases stress tolerance under low-light conditions and improves photosynthesis^22^, and the other protein was the probable Plastid lipid-Associated Protein PAP4, which plays a role in heat stress responses of chloroplast genes with an iron containing superoxide dismutase, decreasing oxidative stress from hydrogen peroxide^23^.

In summary, these proteins are playing key roles as factors in ripening and senescence, and serve as potential targets for prolonging shelf life by interacting with proteins that act as activators or repressors in the senescence process of passion fruit. It also indicates the stress the fruit is under as well as the subsequent mechanisms used to counteract lipid accumulation, heat, and oxidation.

### Functional insights from gene ontology analysis of DEPs in passion fruit tissues

A gene ontology analysis was performed to further extrapolate the function of the differentially expressed proteins (Fig. 1C–F). This gives insight into the biological processes, cellular components, and molecular functions that are active within distinct fruit tissue types after harvest and that may serve as indicators of the length of the shelf life, the process of ripening, and the processes that contribute to preserving quality over time. All four tissue types had enrichment in pathways relating to carbon metabolism, photosynthesis, and secondary metabolite biosynthesis.

For the epicarp, the main biological process was carbon fixation, carotenoid biosynthesis, photosynthesis, and pyruvate metabolism (Fig. 1C). The enrichment of these pathways reflects the nature of the epicarp as the layer that interacts with the outside environment and is a source tissue for anthocyanin and carotenoid synthesis through carbon fixation^2^, and for the accumulation of sugars to later be transported to produce pulp through photosynthesis. The oxidative phosphorylation pathway, or ATP synthesis coupled to the movement of electrons through the mitochondrial electron transport chain at the expense of oxygen, was also enriched in the passion fruit epicarp. In strawberry, oxidative phosphorylation was found to have an important role in the onset of ripening^24^ and is predicted to produce the motive force (ATP and NADPH) for metabolic reactions. Along with the process of de-greening, protein/DNA/RNA turnover occurs, sugar metabolism increases through glycolysis, sucrose turnover, and starch, pectin, and cellulase as well as the increase in flavonoid metabolism are involved in lignin synthesis. It is important to note that half of the differentially expressed proteins in the epicarp were upregulated, while the same genes were downregulated in the other three tissue types. It is possible that the upregulation of these biological processes in the epicarp act as inhibiting processes in the mesocarp and endocarp to initiate degradation.

In the mesocarp, the key pathways included taurine and hypotaurine metabolism, nitrogen metabolism, terpenoid backbone biosynthesis and carbon fixation (Fig. 1D). It is important to note that the differentially expressed proteins in these pathways were downregulated. Taurine, hypotaurine, and terpenoids are oxygen scavengers, and the downregulation of their biosynthesis may lead to increased oxidative stress. In grape, the ROS scavenger hypotaurine delays postharvest softening by regulating pectin and cell wall metabolism pathways^25^. It remains unknown what causes the downregulation of this antioxidant.

In the endocarp, taurine and hypotaurine metabolism were significantly enriched (similar to the mesocarp) along with linoleic acid metabolism, flavonoid biosynthesis, photosynthesis, and alanine, aspartate and glutamate metabolism (Fig. 1E). There were more biosynthetic pathways downregulated in the endocarp. Linolic acid is a monounsaturated fatty acid, and the ripening and lipid peroxidation often occurs simultaneously. The oxidative attack on lipids is the core reaction of ferroptosis. Uncontrolled lipid peroxidation and the production of lipid peroxyl radicals, hydroperoxides, and various oxidation products lead to membrane rupture and cell death^26^. In this study, the production of antioxidants is upregulated in the epicarp, implying that oxidative stress is a key factor during fruit ripening and that senescence is associated with oxidative mechanisms^27^. Passion fruit is also a source of fatty acids, where the accumulation leads to oxidation, resulting in a change in flavor (formation of off-flavor) and the breakdown of cell membranes, which leads to fruit decay. The degradation of lipids occurs through processes such as autoxidation, photooxidation, and enzymatic oxidation and produces a wide variation of volatile compounds. The oxidation of unsaturated fatty acids leads to the generation of hydroperoxides that break down odor-active volatile secondary lipid oxidation products including aldehydes, alcohols, and ketones^28^. This shows the importance of antioxidants as necessary targets for breeding passion fruit varieties with delayed fruit decay to keep quality, nutrition, and flavor at the maximum potential for an extended amount of time.

In the pulp, the most significant pathways with enriched DEPs included those pertaining to fructose metabolism, falvin mononucleotide (FMN) adenylyltransferase activity, glyceraldehyde-3-phosphate dehydrogenase activity, and photosynthesis (Fig. 1F). A potential limitation of using p values and enrichment folds with high stringency as is popular in statistics is a loss of information that is not statistically significant but with important biological functions. In this study, the reason for a lower amount of differentially expressed proteins in the pulp would be that the proteins are more similar in value to other tissue types causing them to not be significantly different with a p value < 0.05 when compared to the universal control that is a mixture of all samples. Carbon metabolism is linked to the ripening process where sucrose degradation accompanied by increases in glucose and fructose levels^29^. Passion fruit is a climacteric fruit, with ethylene controlling the ripening process through increased respiration and changes in the chemical composition and physical characteristics of the fruit. This increase in respiration expedites the water loss in the fruit, drying the pulp and fruit skin. The volatile precursors of ethylene are vital components of the unique flavor passion fruit. Carbon metabolism in the epicarp and carbon fixation in the mesocarp and endocarp all play important roles in the ripening process, and the accumulation of sugars, organic acids, and fermentation products help determine the final fruit quality.

### Trends in upregulated and downregulated tissue specific responses and implications for postharvest quality

There were 213 proteins that showed a fold increase greater than 1.45 or a fold decrease less than 0.45 (at *p* < 0.05). Of this subset, 44 proteins in the epicarp and one protein in the mesocarp were upregulated (Table 1). The rest of the proteins were downregulated in their respective tissues. These upregulated proteins were related to photosynthesis, oxidative phosphorylation, and carbon metabolism. The top proteins with the greatest enrichment included PGR5-like A, the senescence-specific cysteine protease SAG12, non-specific lipid-transfer proteins, peroxidase, ferredoxin, and the pathogenesis-related thaumatin superfamily protein.

**Table 1:** Significantly upregulated and downregulated proteins in four passion fruit tissues.

Tissue type	Upregulated	Downregulated	Total
Epicarp	49	64	113
Mesocarp	1	58	57
Endocarp	0	118	118
Pulp	0	5	5

A total of 213 differentially expressed proteins separated by tissue type that showed a fold increase greater than 1.45 or a fold decrease less than 0.45 (*p* < 0.05).

Lipids are regulators of cellular senescence, and glycolipid and fatty acid turnover occur during this time, although it is not widely studied^30^. This data shows that a major component of postharvest senescence in passion fruit is SAG12, which may have a developmental senescence-specific cell death function during apoptosis, heavy metal detoxification, and hypersensitive response.

The downregulated proteins in the epicarp were related to glycosphingolipid biosynthesis, carotenoid biosynthesis, carbon fixation, pyruvate metabolism, cysteine and methionine metabolism, carbon metabolism, protein processing in the endoplasmic reticulum, and biosynthesis of secondary metabolites. Interestingly, glycosphingolipid and carotenoid biosynthesis were downregulated, while the transporters and oxidative damage proteins were upregulated. The roles of glycosphingolipids in senescence have been widely studied in animals, but not in plants. In yeast and animal cells, sphingolipids are involved in cellular responses, including aspects of signaling, apoptosis, and senescence^31^. This is a potential biosynthesis pathway to study further in passion fruit when it comes to working towards extending the shelf life.

**Figure 2:**
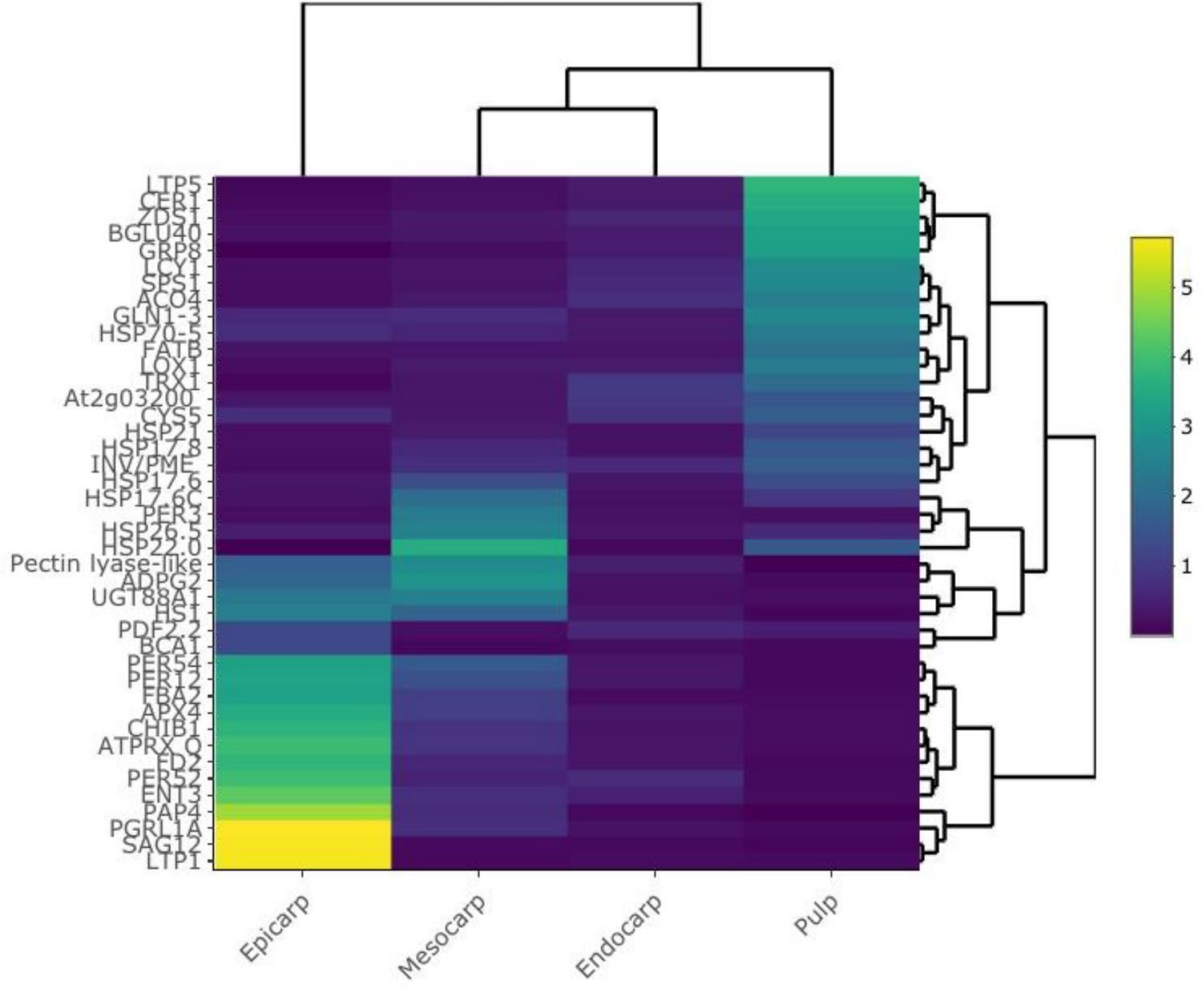
Heatmap of differentially expressed proteins potentially related to shelf life.

Fruit ripening involves many metabolic changes associated with complex and intertwined physiological and biochemical processes. Many genes regulate metabolite biosynthesis, and related cellular responses are up- or down-regulated during passion fruit ripening. Thus, a comparative investigation using distinct tissues with different flesh texture types should provide insight into the mechanisms underlying the variation in the ripening process. Our proteomics study revealed that the distinct tissues of the passion fruit—outer layers and pulp – showed distinct protein profiles (Fig. 2). The epicarp had the largest number of upregulated DEPs, while the DEPs in the other tissues mainly were downregulated. This may reflect a distinct response to ethylene and other critical physiological and environmental cues during the passion fruit ripening process.

### Key proteins in postharvest passion fruit tissues: insights into ripening, senescence, and stress response mechanisms

**Figure 3:**
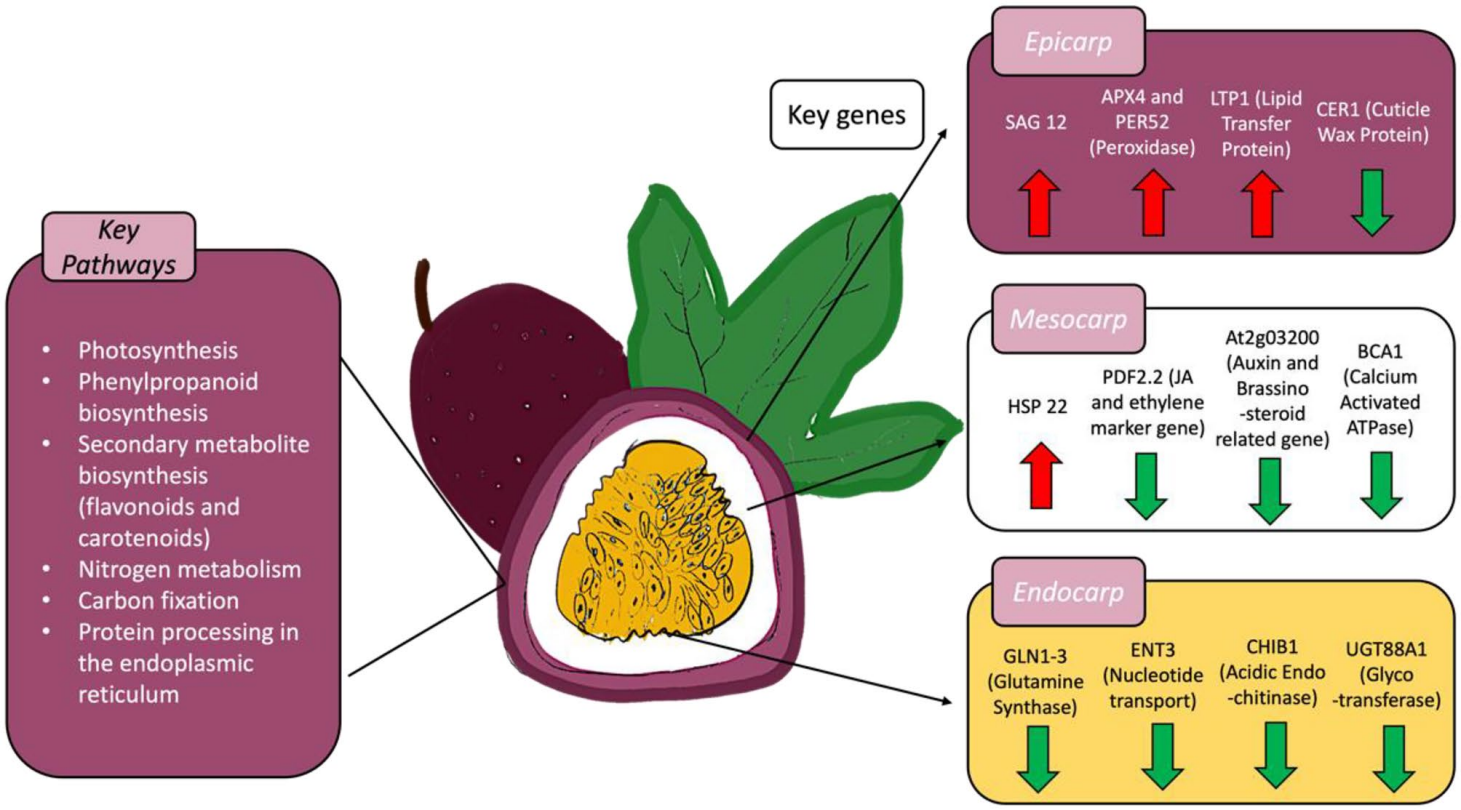
A schematic of passion fruit tissue-specific key up- and down-regulated genes. Up-regulated genes were highlighted in red arrows, down-regulated genes were highlighted in green arrows.

Among the 213 significant DEPs, there was a diverse set of proteins within the four tissue types that may serve key roles in ripening and contribute to the short shelf life of purple passion fruit. Some key differentially regulated processes included cuticle wax formation, hormone signaling pathways (ethylene, jasmonic acid, and gibberellic acid), heat shock proteins, stress tolerance proteins, antioxidant activity, cell wall metabolism, lipid metabolism and secondary metabolite biosynthesis (Fig. 3). In addition, two homologs were found to proteins (TXND9 and PLAC8) that are currently being researched for their potential as targets for gene therapy in cancerous growths^32,33^.

The first protein of interest, ECERIFERUM 1 (CER1), was downregulated in the epicarp and mesocarp. CER1 is a fatty acid hydroxylase that has been identified as a cuticle wax protein in a QTL study in citrus^34^. Plant aerial organs are coated with cuticular waxes, serving as a hydrophobic water-proofing barrier. This wax is a mixture of aliphatic long-chain molecules ranging from 22 to 48 carbons that are produced in the endoplasmic reticulum of epidermal cells, and alkanes represent up to 80% of the total wax^35^. From a postharvest perspective, CER1 would affect water loss, and fewer alkanes in the cuticular wax could reduce storability.

The hormone signaling pathways for ethylene, jasmonic acid (JA), and gibberellic acid (GA) were differentially expressed in this study. In the ethylene pathway, ACC oxidase 4 (ACO4) was downregulated in the passion fruit epicarp, potentially signifying the onset of senescence in this tissue. In plants, ethylene is biosynthesized from S-adenosyl-L-methionine (SAM) through the intermediate 1-aminocycloprone-1-carboxylic acid (ACC). Then, the conversion of ACC to ethylene is done by the ACC oxidases (ACOs). The onset of climacteric ripening corresponds to the transition from autoinhibitory to autocatalytic ethylene biosynthesis and was reported to be associated with dramatic upregulation of ACS2, ACS4, ACO1, and ACO4, resulting in positive feedback regulation^36^. Within the jasmonic acid pathway, LOX1, TET8, and PDF2.2 were downregulated in the epicarp and mesocarp. LOX1 (linoleate 9S-lipoxygenase 1) participates in the production of hydroperoxide derivatives from polyunsaturated acids and regulatory development by controlling the biosynthesis of aroma volatiles, which is stimulated by maturity and involved in the regulation of jasmonic acid^37^. JA has been previously found to delay weight loss in plums, extend the shelf life in papaya, and extend shelf life and improve postharvest quality in fruit^38^. TET8 (tetraspanin 8) is involved in the JA signaling pathway in response to mechanical wounding^39^. PDF2.2 is an ethylene and jasmonic acid marker gene^40^. The gibberellin-regulated protein 8 (GRP8) was downregulated in passion fruit. A decrease in GRP8 would cause a reduction in gibberellic acid (GA) synthesis. GAs promote fruit enlargement and coloration as well as maintain fresh fruit quality^41^.

Several heat shock proteins, HSP17.8, HSP70-5, HSP26.5, HSP16.6C, and HSP21, were downregulated in the mesocarp, while HSP 22 was upregulated in this tissue (Fig. 3). HSP22 is a small ubiquitous heat shock protein whose expression is induced in response to a wide variety of unfavorable physiological and environmental conditions^42^. Heat shock proteins protect cells from lethal conditions because of their involvement in cell death pathways such as necrosis, apoptosis, or autophagy. Cell death is avoided by the role of HSPs in preventing denatured proteins from aggregating, regulation of caspase activity, regulation of the intracellular redox state, actin polymerization and maintaining cytoskeleton integrity, and proteasome-mediated degradation of selected proteins^42^.

The levels of cell wall proteins such as pectin lyase were differentially regulated in passion fruit. Ectopic expression of a pectin-degrading enzyme leads to the release of cell wall epitopes that serve as signals for defense gene activation^43^. Moreover, plant invertase/pectin methylesterase inhibitor superfamily proteins are crucial enzymes for coordinating carbohydrate metabolism, stress responses, and sugar signaling. Invertases catalyze the cleavage of sucrose into glucose and fructose, exerting a pivotal role in sucrose metabolism, cellulose biosynthesis, nitrogen uptake, and reactive oxygen species scavenging as well as osmotic stress adaptation. PMEs exert a dynamic control of pectin methyl esterification to manage cell adhesion, cell wall porosity, and elasticity, as well as perception and signaling of stresses^44^. In addition, PER3 is a peroxidase enzyme involved in using hydrogen peroxide and other substrates as oxygen donors. PER3 was also found to be in the crucial last step of lignin biosynthesis in the phenylpropanoid biosynthesis pathway^45^.

The levels of nonspecific lipid proteins such as LTP1 varied among the passion fruit tissues (Fig. 3). PAP13, PAP4, LTP5, and LTP10 were also found to be differentially expressed in the dataset. LTPs are small, cysteine-rich proteins that play numerous functional roles in plant growth and development, including cutin wax formation, pollen tube adhesion, cell expansion, seed development, germination, and adaptation to changing environmental conditions^46^. They also control immune-related responses in plant-pathogen interactions and regulate metabolism by signaling the control of homeostasis. LTPs are widely known for their antimicrobial activities considering their strategy in disturbing pathogens’ membrane integrity^47^, as well as their influence on cutin wax formation, cell wall organization, signal transduction^48^, and antioxidant activity^49^ may impact the shelf life in fruit.

### Key pathways governing postharvest passion fruit

**Figure 4:**
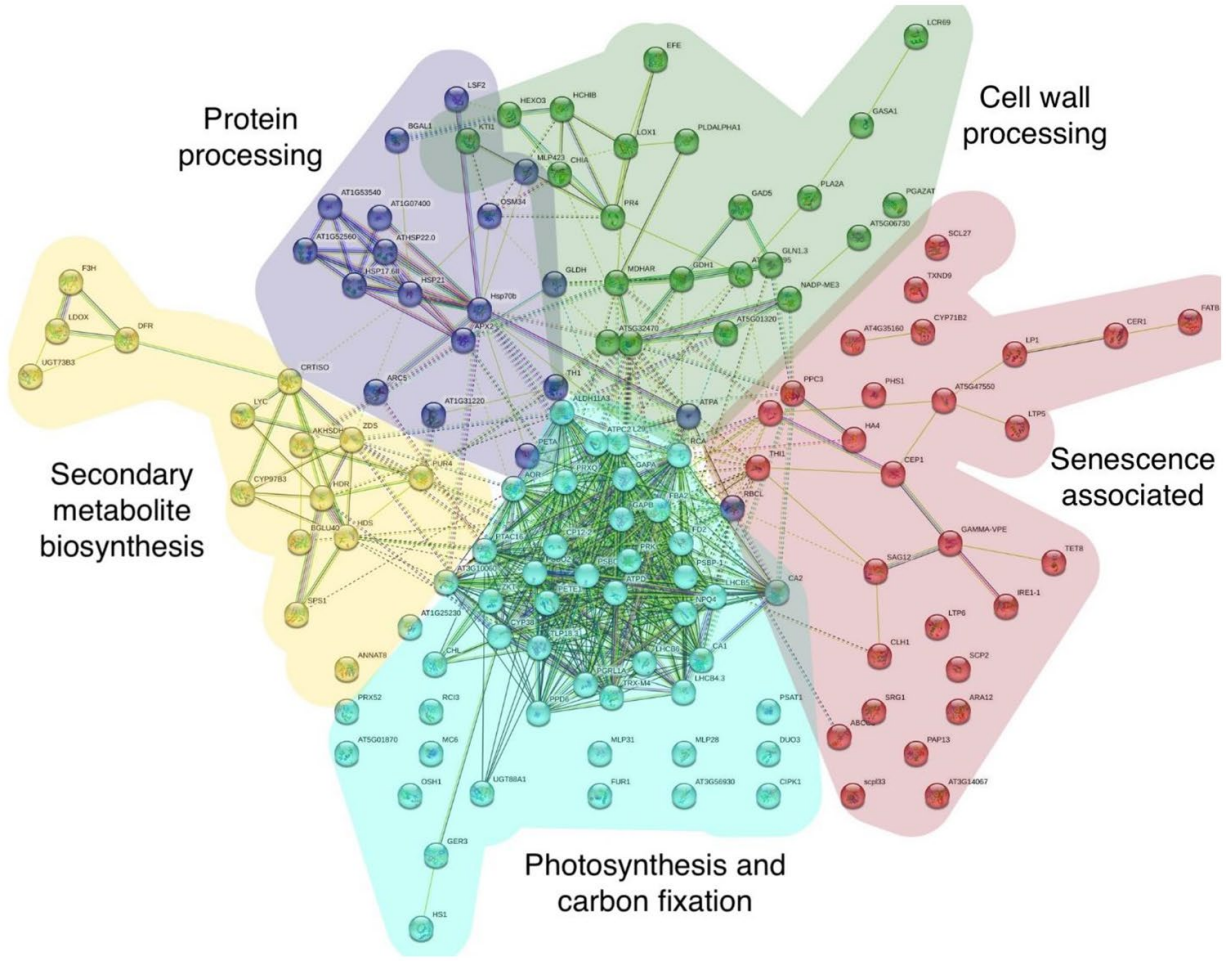
Protein–protein interaction network created with STRING and k-means clustering. The color of the nodes represents KEGG Pathway.

Using the KEGG database via the PaintOmics4 online tool, 122 pathways were found for the entire proteome database. Of them 74.59% of the pathways were related to metabolism (i.e. amino acids, carbohydrates, secondary metabolites, lipids, etc.), 16.39% were related to genetic information processing, 3.28% to cellular processes (transport and catabolism), as well as for environmental information processing (signal transduction and membrane transport), and 1.64% of proteins related to an unspecified category. Out of the 122 pathways identified in this study, nine pathways were statistically significant using a combined p-value < 0.05 in Fisher’s test (Fig. 4). Protein–protein interactions were mapped using the STRING database (Supplementary Table S2) and shown potential interactions within these biological function pathways (Fig. 4). Protein clusters are detailed in Supplementary Table S3. Two of these pathways include the endoplasmic reticulum associated degradation (ERAD) and the phenylpropanoid pathway are highlighted in the following paragraphs.

The endoplasmic reticulum (ER) is an important subcellular organelle and serves as a checkpoint for protein folding. It has an essential role in ensuring the proper folding and maturation of newly secreted and transmembrane proteins^50^. Some of the processes that occur here are glycosylation, folding, and/or the assembling of these proteins into protein complexes. This is an error-prone process and it is possible for there to be an accumulation of unfolded or misfolded proteins causing stress in the ER. ER-associated degradation and autophagy are strategies to account for this stress^51^. This pathway was significant in the passion fruit proteome and may be an important aspect for shelf life because the downregulation of this vital process that upkeeps proteins could lead to a cascade effect resulting in cell death and fruit decay.

The phenylpropanoid pathway was a pathway that was significant in this dataset. This pathway plays an important role in the enhanced aroma and flavor of fruit^52^. It is a precursor for non-volatile and volatile metabolites. The non-volatile metabolites include lignin, flavonoids, anthocyanins, glycosylates, ubiquinone, and more. The volatile compounds are C6-C2 compounds, benzoids, and phenylpropanoids, GABA treatment can prolong the shelf life and delay the senescence of the fruit by regulating the phenylpropanoid pathway as well as reactive oxygen species metabolism^53^. This is an interesting pathway to target in plant breeding because the non-volatile compounds are precursors for cell wall components that may increase firmness and shelf life, while the volatile compounds would maintain the flavor profile.

### Characterization of hypothetical proteins: cell wall metabolism, hormone homeostasis, and senescence

**Figure 5:**
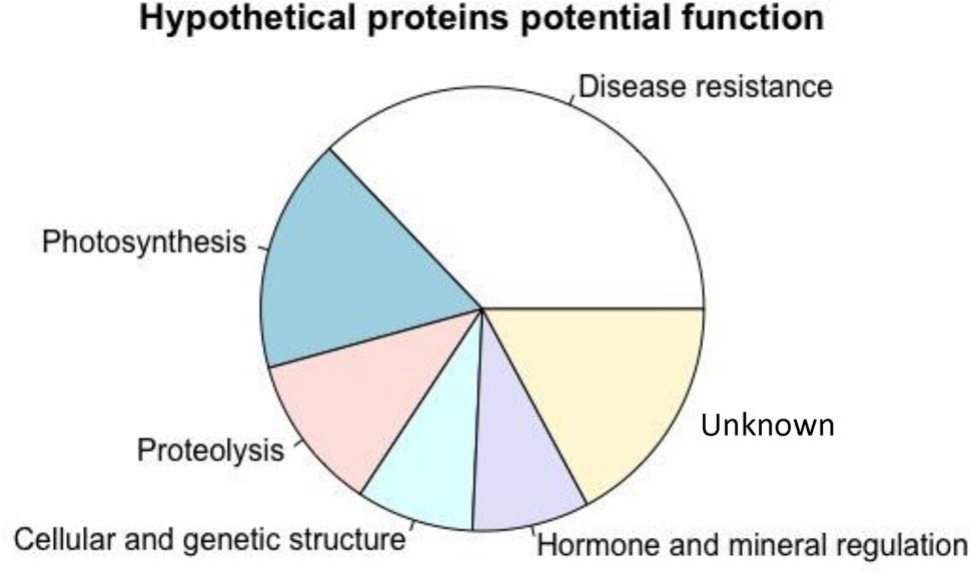
Hypothetical proteins predicted function.

The proteomics analysis identified 3352 proteins, and each was mapped to known *Arabidopsis thaliana* homologs. Out of 295 differentially expressed proteins (DEPs), 36 proteins did not match any known *A. thaliana* protein and were characterized as hypothetical proteins (Fig. 5 and Table 2). These proteins were characterized using position-specific iterative basic local alignment search tool (PSI-BLAST) described in the methods section. Those proteins can be categorized into six major functional groups including ‘disease resistance’, ‘photosynthesis’, ‘proteolysis’, ‘cellular and genetic structure’, ‘hormone and mineral regulation’, and ‘unknown’ (Fig. 5). We listed those proteins in Table 2 and summarized their predicted functions below.

**Table 2:** Homolog gene function, species, and percent identity serve as the characterization of 36 hypothetical proteins in the passion fruit skin layer tissues.

Protein ID	*Passiflora* tissue	Homologous gene	Function	Homolog species	Percent identity
ZX.01G0036000	Endocarp	Chlorophyll a-b binding protein	Photosynthesis	Pepper	67.77%
ZX.01G0038500	Endocarp	Mavicyanin-like	Photosynthesis	Pink shepherd’spurse	37.89%
ZX.01G0136920	Epicarp and endocarp	psbP-like protein 1	Photosynthesis	Riverbank grape	55.78%
ZX.04G0000820	Endocarp	Umecyanin-like	Photosynthesis	Silver poplar	35.16%
ZX.01G0136920	Epicarp and endocarp	psbP-like protein 1	Photosynthesis	Riverbank grape	55.78%
ZX.01G0069330	Epicarp and endocarp	Kinesin-like protein KIN-14K	Immunity	Sweet orange	51.33%
ZX.08G0034570	Mesocarp and endocarp	Natterin-3 like	Immunity	Kiwi	
ZX.08G0040150	Epicarp and endocarp	STH-21-like	Immunity	Pomegranate	46.63%
ZX.08G0040080	Epicarp and mesocarp	Major allergen Pru ar 1-like	Immunity	Chardonnay	41.14%
ZX.08G0040090	Epicarp	Major allergen Pru ar 1-like	Immunity	Riverbank grape	48.39%
ZX.08G0040080	Epicarp and mesocarp	Major allergen Pru ar 1	Immunity	Chardonnay	41.77%
ZX.08G0040150	Epicarp and endocarp	Pathogenesis-related protein STH-21-like	Immunity	Pomegranate	46.63%
ZX.01G0076680	Mesocarp	Lipid-transfer protein	Immunity	Cacao tree	61.76%
ZX.08G0027000	Mesocarp	Miraculin-like	Immunity	Passion fruit	98.76%
ZX.08G0027030	Mesocarp	Miraculin like	Immunity	Passion fruit	98.76%
ZX.08G0031700	Mesocarp	Miraculin like	Immunity	Passion fruit	54.21%
ZX.08G0031750	Mesocarp	Miraculin like	Immunity	Passion fruit	45.32%
ZX.08G0031800	Mesocarp	Miraculin like	Immunity	Passion fruit	53.60%
ZX.08G0031830	Mesocarp	Miraculin like	Immunity	Passion fruit	49.60%
ZX.08G0034570	Mesocarp and endocarp	Natterin 3-like	Immunity	Kiwi	54.00%
ZX.01G0055410	Epicarp and endocarp	Zinc transporter-like	Homeostasis	Kiwi	55.64%
ZX.01G0055410	Epicarp and endocarp	Zinc transporter-like	Homeostasis	Kiwi	32.63%
ZX.04G0033240	Epicarp	Embryonic protein DC-8	Homeostasis	Lettuce	18.26%
ZX.09G0005160	Epicarp and mesocarp	Heterodimeric geranylgeranyl pyrophosphate synthase	Homeostasis	Cork oak	57.84%
ZX.07G0017660	Mesocarp	S-norcoclaurine synthase 2-like	Homeostasis	Cowpea	46.58%
ZX.09G0005160	Epicarp and mesocarp	Heterodimeric geranylgeranyl pyrophosphate synthase	Homeostasis	Cork oak	57.84%
ZX.01G0060170	Endocarp	Chitinase 2-like	Cell structure	Wild Colombian Cacao	84.44%
ZX.01G0014300	Epicarp and endocarp	Curvature thylakoid 1A	Cell structure	White poplar	60.90%
ZX.08G0028880	Mesocarp and endocarp	Kinesin kp1	Cell structure	Wild tobacco	
ZX.01G0014300	Epicarp and endocarp	Glutamyl-tRNA synthetase	Protein metabolism	Asparagus bean	75.81%
ZX.09G0014090	Epicarp and endocarp	NAC domain-containing protein 78, putative isoform 1	Protein metabolism	Cacao tree	30.25%
ZX.01G0067000	Mesocarp	Multicystatin-like	Protein metabolism	Wild potato	32.53%
ZX.08G0028930	Endocarp	Nuclease SbcCD subunit C-like	RNA and DNA repair	Silver poplar	44.32%
ZX.00003010	Mesocarp	DEAD/DEAH box helicase	RNA and DNA repair	Gram positive bacteria	78.78%
ZX.01G0115610	Endocarp	Plastid-lipid-associated protein 6	Senescence	Physic nut	0.002608651
ZX.09G0014090	Epicarp and endocarp	NTM1-like 9 isoform X3	Senescence	New South Wales waratah	2.60E-05

#### Proteins involved in photosynthesis

Five proteins were initially labeled as unknown proteins from the harvested passion fruit and later were found to be involved in photosynthesis (Table 2). Chlorophyll a-b binding protein functions as a light receptor and captures and delivers energy to photosystems and binds at least 14 chlorophylls and carotenoids such as lutein and neoxanthin^54^. All of these proteins were found in the endocarp layer and may indicate a tissue-specific function of these proteins.

#### Proteins involved in plant immunity

There were two natterin-3-like proteins found in the epicarp and endocarp. These were first discovered in the venom of the medically significant fish *Thalassophyrne nattereri* and have since then been found in various organisms performing immune-related functions. They have been found to activate multiprotein complexes driving the host’s innate immune responses and have been found to be involved in embryogenesis where a CRISPR knockout caused mutations in growth retardation, spinal defects, heart dysfunction, and more^55^. Three major pru ar 1-like proteins were identified in passion fruit, which may be the major cause of allergies upon the fruit consumption. These proteins are part of the abscisic acid binding pathway, protein phosphate inhibits activity, signaling receptor activity, defense response, and response to biotic stimulus and may be a pathogenic response to an attack in passion fruit^56^. Six miraculin-like proteins were found in the mesocarp and have been previously characterized in studies on gene expression exposure to be differentially expressed and downregulated in response to canker disease. They are homologous to miraculin which is a homodimer protein with a taste-modifying activity that converts sourness into sweetness^57^. Canker is one of the leading causes of vine death in Florida and is a major issue to growers. These miraculin-like proteins in passion fruit may confer potential resistance to pathogenic attacks. Two STH-21-like proteins were found which are part of a class of proteins related to biotic and abiotic stress to protect plants against damage. It is anti-fungal and has RNase activity^58^. The expression of this gene is induced by several signaling molecules, including methyl jasmonate, ethephon, hydrogen peroxide, and salicylic acid, and was found in both the epicarp and endocarp. Kinase-like protein KIN14k was also found in both the endocarp and epicarp and supports microtubule movement in an ATP-dependent manner. They are important for male and female meiosis for gametophyte development^59^. A lipid transfer protein was also found in the mesocarp and involved with lipid metabolism^60^.

#### Proteins involved in the homeostasis of zinc and magnesium

Two proteins with 55% and 32.63% similarity to Zinc-Induced Facilitator 1 (ZIFL1) were present in the epicarp and endocarp and reported to regulate the zinc levels by controlling the zinc influx and efflux between the extracellular and intracellular compartments and thus modulating the zinc concentration and distribution^61^. Two heterodimeric geranylgeranyl pyrophosphate synthase small subunit, chloroplast-like were found in the epicarp and mesocarp, and it is a small subunit that is inactive alone while the large subunit GGPPS1 catalyzes the main production of geranyl-diphosphate in vitro. The association of the two subunits will change the product profile and increase the production of geranyl-diphosphate. Every subunit binds to two Mg^2+^ which act as cofactors in this process. It is a chloroplast thylakoid membrane with metal ion binding and is part of the monoterpene biosynthetic process. Interestingly, S-norcolaurine synthase 2-like was found and is the first step in the biosynthesis of benzylisoquinoline alkaloids with medicinal and drug properties such as it is used in morphine^62^. This may serve as a pathogen-related protein and is involved in the monoterpene biosynthesis of myrcene. Embryonic protein DC-8 was present in the epicarp and regulates abscisic acid, it is a protein located in the cytoplasm and cell walls studied in carrot embryos and endosperm^63^.

#### Proteins involved in cell wall and chloroplast structures

Chitinase 2-like protein was downregulated in the endocarp and it is responsible for the creation of the cell wall via carbohydrate metabolism. It has been found to be required for the proper cell wall biosynthesis in etiolated seedlings and prevents lignin accumulation in the hypocotyls^64^. It is located in the extracellular region for the carbohydrate metabolic process for the cell wall, chitin catabolic process, and carb binding. This serves as a potentially novel candidate protein for extending the shelf life of passion fruit if targeted in the epicarp. Curvature thylakoid chloroplast-like protein was located in the epicarp and endocarp and is part of modifying thylakoid architecture by inducing membrane curvature^65^.

Kinesin kp1 in the mesocarp and endocarp is predicted to be a plant specific motor protein with transport function and is plant specific. It constitutes a superfamily of ATP-driven microtubule motor enzymes that convert the chemical energy of ATP hydrolysis into mechanical work along microtubule tracks. The diverse cellular functions of this superfamily are microtubule dynamics and morphogenesis, chromosome segregation, spindle formation, and elongation and transport of organelles^66^.

#### Proteins involved in protein metabolism

Glutamyl-tRNA synthetase is involved in protein synthesis and activates glutamine by covalently linking to a specific amino acid to the correct tRNA for protein synthesis. Prior binding of the tRNA is required for the activation of glutamine by ATP and these proteins were differentially expressed in the epicarp and endocarp^67^. NAC- domain-containing protein 78 was in the epicarp and endocarp and is responsible for the proteolysis of degraded cells as part of cellular housekeeping. It is a transcriptional activator activated by proteolytic cleavage through regulated intermembrane proteolysis and responsible for stress responses by removing abnormal or misfolded proteins, supplying amino acids needed to make new proteins, assisting in the maturation of zymogens and peptide hormones by limited cleavages, controlling metabolism, homeosis, and development by reducing the abundance of key enzymes and regulatory proteins, and for programmed cell death of specific plant organs or cells^68,69^. Multicystatin-like protein in the mesocarp regulates protein catalysis and is composed uniquely of eight repeating units, each capable of inhibiting cysteine proteases. It has defense-related implications in tuber physiology via its ability to regulate protein catabolism^70^.

#### Proteins involved in RNA/DNA repair

Nuclease SbcCD subunit-like protein in the endocarp can be influencing the structural maintenance of chromosomes. This protein cleaves hairpin structures that halt the progress of the replication form, allowing homologous recombination to restore DNA replication^71^. DEAD/DEAH box is related to RNA metabolism. These enzymes are involved in processes that require the manipulation of RNA structures with highly conserved RNA binding proteins and ATPase activity. It has been studied in the human genome relating to the regulation of embryonic development, cell proliferation, hematopoiesis, metabolism, innate immunity and immune programming, cancer pathogenesis, inflammation, and autoimmune disease and used as therapeutic gene targets^72^.

#### Proteins involved in senescence

Plastid lipid-associated protein in the endocarp is involved in repair, metabolism, stress response, and senescence. It is chloroplastic and required for plastoglobuli development and resistance to multiple stresses and osmiophilic content. It may be involved in the transport of lipophilic antioxidants in and out of the plastoglobuli^73^. NTM1-like 9 isoforms are involved in immune defense and senescence in the epicarp and endocarp. This protein is a NAC transcription factor and is a calmodulin that regulates transcriptional repressor and acts as a positive regulator of immunity involved in effector-triggered immunity (ETI) induction of immunity-related gene expression as well as mediates osmotic stress signaling in leaf senescence by up-regulating senescence-associated genes^74,75^.

## Conclusion

In conclusion, our study on tissue-specific proteome profile analysis has provided valuable insights into the regulatory and stress-responsive networks operating in passion fruit during storage. The consumer acceptance and shelf life of fresh passion fruit are contingent on the preservation of an intact and firm epicarp without pathogenic blemishes. By investigating the underlying biological mechanisms governing texture transformation during ripening, we have shed light on crucial factors that can influence the storability of fresh fruit.

Utilizing the TMT technique to tag proteins from multiple tissue samples, we identified 295 differentially expressed proteins (DEPs), with 50 proteins exhibiting upregulation in specific tissues. During the ripening process, these DEPs primarily play roles in energy production and conversion, as well as in the metabolism of carbohydrates, amino acids, and lipids. Notably, the key upregulated proteins in the epicarp are associated with photosynthesis, oxidative phosphorylation, and carbon metabolism, while downregulated proteins in the epicarp and other tissues are linked to various pathways such as glycosphingolipid biosynthesis, carotenoid biosynthesis, and protein processing in the endoplasmic reticulum, among others.

Additionally, we used bioinformatics tools to characterize 36 hypothetical proteins based on sequence and structure analyses. These proteins represent potential novel candidate genes in passion fruit, implicated in crucial processes such as photosynthesis, immunity, homeostasis, cell structure, protein metabolism, RNA and DNA repair, and senescence pathways.

In summary, our findings provide a comprehensive understanding of the functional roles of DEPs in controlling passion fruit development and quality during ripening and postharvest. This dataset serves as a foundational platform for further investigations into the functions of these proteins and pathways, paving the way for future improvements in fruit quality traits and the overall enhancement of passion fruit as a consumer product.

## Methods

### Plant materials

Purple passion fruits from mature vines of *Passiflora edulis* (Sims) were obtained at full maturity from the University of Florida Tropical Research and Education Center in Homestead, Florida and transported to Gainesville, Florida. Fully ripe fruit were kept at room temperature (RT), and the tissue layers were separated using a scalpel within 5 days after harvesting. Samples were frozen in aluminum foil in liquid nitrogen and stored at – 80 °C until protein extraction. Four replicates were used for the mesocarp, endocarp, and pulp and two replicates were used for the epicarp. Experimental research and field studies on all plant samples, including the collection of plant materials, complies with relevant institutional, national, and international guidelines and legislation.

### Protein extraction, digestion, and tandem mass tagging

Tissues of the passion fruits were dissected as epicarp, mesocarp, endocarp, and pulp. Each tissue was processed for protein extraction according to Fujiki et al.^76^, with the following modifications. Briefly, samples were ground in liquid nitrogen into a fine powder and incubated in extraction buffer (0.1 M Tris–HCl pH 8.8, 10 mM EDTA, 0.2 M DTT, 0.9 M sucrose) with continued grinding in a fume hood. The extract was then agitated for 2 h at RT. After washing twice with 0.1 M ammonium acetate in methanol and twice with 80% acetone, the dried pellet was dissolved with 50 mM ammonium bicarbonate buffer. Each suspension was incubated on ice before centrifugation (4 °C) for 20 min at 12,000×*g*. The soluble proteins were quantified using an EZQ Protein Quantitation Kit (Invitrogen, Carlsbad, CA, USA) with the SoftMax Pro Software v5.3 (Molecular Devices, Downingtown, PA, USA). Proteins were dissolved in protein buffer [8 M Urea, 50 mM Tris–HCl, pH 8.0, 50 mM triethylammonium bicarbonate, 0.1% SDS (w/v), 1% Triton-100 (w/v), 1 mM PMSF, 10 μg/ml leupeptin, 1% phosphatase inhibitor cocktail 2 and 3 (v/v)]. For each sample, a total of 200 μg of protein were reduced with 10 mM tris(2-carboxyethyl)phosphine, alkylated with 20 mM iodoacetamide, trypsin-digested (w/w for enzyme:sample = 1:100), and labeled according to the manufacturer’s instructions (Thermo Scientific, Rockford, IL, USA). Epicarp extracts were labeled with TMT tags 126, and 128C, mesocarp extracts were labeled with TMT tags 127N, 129N, 130C, and 132N, endocarp extracts were labeled with 127C, 129C, 131N, and 132C, and pulp extracts were labeled with 128N, 130N, 131C, and 133N. Therefore, all experiments were performed with biological duplicates or quadruplicates per group.

Additionally, aliquots of all sample mixtures were labeled with TMT tags 133C and 134N as the control group. The universal control used in this experiment is a mixture of all samples as standard protocol. Each tissue type was individually compared to the universal control with a t-test with significantly expressed proteins designated as those with a p value < 0.05 and a significant enrichment fold cut off of < 0.45 for downregulated proteins and > 1.45 for upregulated proteins when compared to the control. This high stringency allows for proteins that are all at similar values to in the tissue types, when compared to the control is not significant. Those that are vastly lower or higher than the control will be identified as significant.

### Basic pH reversed-phase fractionation and LC–MS/MS analysis

Labeled peptides were desalted with C18-solid phase extraction and dissolved in basic pH reversed-phase (bRP) buffer A (10 mM NH_4_HCO_2_, pH 10, 5% ACN). The peptides were fractionated using an Agilent HPLC 1260 with a Zorbax Extended C18 bRP column (2.1 × 150 mm, 5 μm, Agilent, Santa Clara, CA) using a gradient of 10–40% bRP buffer B (10 mM NH_4_HCO_2_, pH 10, 90% ACN) over 50 min by ramping up to 100% solvent B in 5 min at a flow rate of 200 μl∙min^-1^. The absorbance at 280 nm was monitored, and a total of 19 fractions were collected. The fractions were lyophilized, desalted using zip tips (Millipore C18 Zip Tips, Billerica, MA, USA), and resuspended in LC solvent A (0.1% formic acid in 99.9% water, v/v). A hybrid quadrupole Orbitrap Fusion Tribrid MS system (Thermo Fisher Scientific, San Jose, CA, USA) was interfaced with an automated Easy-nLC 1200 system (Thermo Fisher Scientific, Bremen, Germany). Each sample fraction was loaded onto an Acclaim Pepmap 100 pre-column (20 mm × 75 μm; 3 μm-C18) and separated on a PepMap RSLC analytical column (500 mm × 75 μm; 2 μm-C18 100 Å) at a flow rate at 300 nl∙min^-1^ using a linear gradient from 5% solvent A (0.1% formic acid in water) to 25% solvent B (0.1% formic acid and 80.0% acetonitrile) for 110 min, to 40% solvent B for 10 min, to 95% solvent B for 10 min and hold 95% solvent B for additional 10 min. A data-dependent method with multi-notch synchronous precursor selection MS3 scanning was employed. Full MS scans were acquired in the Orbitrap mass analyzer over the 400–1400 m/z range at a set 120,000 resolution. MS2 scans were enabled automatically to isolate fragment precursor ions with charge state filtering from 2 to 6, using collisional-induced dissociation (CID) with a normalized collision energy (NCE) of 35%, an AGC target of 4e5, and a maximum accumulation time of 50 ms. Fragment ions were selected for MS3 scans based on a precursor selection range of 400–1200 m/z, and the top 10 precursors were selected for MS3 scans that were acquired in the Orbitrap after HCD fragmentation (NCE 55%) with a maximum fill time of 120 ms at a set 50,000 resolution, over the 110–500 m/z scan range, with ion injection for all parallelizable time turned OFF, and an AGC target value of 1.2e5. MS1 and MS3 scans were acquired in profile mode, and MS2 in centroid format under a 3-s duty cycle.

### Proteomics data analysis

The raw MS/MS data files were thoroughly compared against the ZX *Passiflora* database (39,101 entries) with consideration to biological modifications and amino acid substitutions using the Proteome Discoverer v2.5 (Thermo Fisher Scientific), with the SEQUEST algorithm^77^. In the study by Ma et al.^13^, the ZX *Passiflora* database was constructed using genomic DNA extracted from passion fruit fresh leaf samples in Xiamen, Fujian Province, China. The Illumina genomic library was used to predict high-quality protein-coding genes for the database using homology-based prediction models compared to eight proteomes (including *Manihot esculenta*, *Ricinus communis*, *Salix purpurea*, *Populus trichocarpa*, *Linum usitatissium*, *Citrus clementeina*, *Arabidopsis thaliana*, and *Oryza sativa).* The following parameters were used for all the searching: peptide tolerance at 10 ppm, tandem MS tolerance at ± 0.02 Da, peptide charges of 2 + to 5 + , trypsin as the enzyme, allowing one missed cleavage, TMT label and carbamidomethyl (C) as fixed modifications, and with acetylation (N-terminus), 2-hydroxyisobutyrylation (K), loss of methionine (N-terminus), oxidation (M) and phosphorylation (S, T, Y) as variable modifications. For peptide confidence, we adopted the following Xcorr cutoff values, which are commonly used for the SEQUEST algorithm^78^: 2.31 for 2 + , 2.41 for 3 + , and 2.6 for 4 + and 5 + peptides. The false discovery rate (FDR) was calculated using the Percolator algorithm in the Proteome Discoverer workflow based on the search results and set at 1%. For protein quantification, only MS/MS spectra that were unique to a particular protein and where the sum of the signal-to-noise ratios for all the peak pairs was > 9 were used. The data were normalized with the total peptide amount and scaled with the average of the control group, and the significance of differential expression was assessed by a *t*-test of p ≤ 0.05 with fold change > 1.55 or < 0.45 (Supplementary Table S4).

### Gene ontology and pathway analysis

ShinyGO v0.77^79^ was used to perform gene ontology enrichment analysis as well as metabolic pathway network enrichment through the Kyoto Encyclopedia of Genes and Genomes (KEGG, www.kegg.jp/kegg/kegg1.html) metabolic pathways collections for *Arabidopsis thaliana*^80^. In addition, the same tool was used for the Search Tool for the Retrieval of Interacting Genes/Proteins for the protein–protein interaction network^81^. The enriched GO term was also used to create a pathway network. In all cases, enrichment studies were statistically tested using Hochberg false discovery rate (FDR) correction and a significance level < 0.05 after correction. PaintOmics4 was used to find the significant metabolic pathways within the proteomic data^82^. Heatmap was created using R package heatmaply. The STRING database was used with k-means clustering to analyze protein–protein interactions.

### Characterization of hypothetical proteins

The protocol for Position-Specific Iterative Basic Local Alignment Search Tool (PSI-BLAST) was following Bhagwat and Aravind^83^, with minor modifications to characterize hypothetical proteins in this study. This process derives a position-specific scoring matrix (PSSM) from the multiple sequence alignment of sequences detected above a given score threshold using a protein–protein BLAST. PSI-BLAST was repeated three times for each protein. This process uncovers distant relationships to predict protein structure folding and functional properties based on similar sequence patterns.

### Supplementary Information

Supplementary Table 1.

Supplementary Table 2.

Supplementary Table 3.

Supplementary Table 4.

## Data Availability

The datasets generated in the analysis of the current study are publicly available in the online repository ProteomeXchange MassIVE partner repository with the data set identifiers PXD044456 and MSV000092624. https://massive.ucsd.edu/ProteoSAFe/dataset.jsp?task=fe601c4bd4cb4251b8169b8c0579237f.
